# Weak Glycolipid Binding of a Microdomain-Tracer Peptide Correlates with Aggregation and Slow Diffusion on Cell Membranes

**DOI:** 10.1371/journal.pone.0051222

**Published:** 2012-12-12

**Authors:** Tim Lauterbach, Manoj Manna, Maria Ruhnow, Yudi Wisantoso, Yaofeng Wang, Artur Matysik, Kamila Oglęcka, Yuguang Mu, Susana Geifman-Shochat, Thorsten Wohland, Rachel Kraut

**Affiliations:** 1 School of Biological Sciences, Nanyang Technological University, Singapore; 2 Institut für Lebensmittel- und Bioverfahrenstechnik, Technische Universität Dresden, Dresden, Germany; 3 Department of Chemistry, National University of Singapore, Singapore; 4 Department of Biological Sciences, National University of Singapore, Singapore; 5 Centre for Bioimaging Sciences, National University of Singapore, Singapore; Emory University School of Medicine, United States of America

## Abstract

Organized assembly or aggregation of sphingolipid-binding ligands, such as certain toxins and pathogens, has been suggested to increase binding affinity of the ligand to the cell membrane and cause membrane reorganization or distortion. Here we show that the diffusion behavior of the fluorescently tagged sphingolipid-interacting peptide probe SBD (Sphingolipid Binding Domain) is altered by modifications in the construction of the peptide sequence that both result in a reduction in binding to ganglioside-containing supported lipid membranes, and at the same time increase aggregation on the cell plasma membrane, but that do not change relative amounts of secondary structural features. We tested the effects of modifying the overall charge and construction of the SBD probe on its binding and diffusion behavior, by Surface Plasmon Resonance (SPR; Biacore) analysis on lipid surfaces, and by Fluorescence Correlation Spectroscopy (FCS) on live cells, respectively. SBD binds preferentially to membranes containing the highly sialylated gangliosides GT1b and GD1a. However, simple charge interactions of the peptide with the negative ganglioside do not appear to be a critical determinant of binding. Rather, an aggregation-suppressing amino acid composition and linker between the fluorophore and the peptide are required for optimum binding of the SBD to ganglioside-containing supported lipid bilayer surfaces, as well as for interaction with the membrane. Interestingly, the strength of interactions with ganglioside-containing artificial membranes is mirrored in the diffusion behavior by FCS on cell membranes, with stronger binders displaying similar characteristic diffusion profiles. Our findings indicate that for aggregation-prone peptides, aggregation occurs upon contact with the cell membrane, and rather than giving a stronger interaction with the membrane, aggregation is accompanied by weaker binding and complex diffusion profiles indicative of heterogeneous diffusion behavior in the probe population.

## Introduction

Numerous pathogenic proteins and viruses utilize sphingolipid-containing domains in the plasma membrane of the target cell as a means of docking and entry into the cell [1]. The dynamics with which sphingolipid-interacting domains found in such proteins engage with their lipid receptors and move in the plane of the membrane has been proposed to involve a weak interaction of aromatic amino acids with carbohydrate-modified glycolipids or acidic gangliosides [2], providing the initial step to a stronger protein receptor-pathogen interaction [3], [4], [5], [6], [7].

Clustering of glycolipids at the membrane by multimerization of ligands is a common mode of interaction by pathogen attachment proteins that increases binding strength and facilitates internalization [2], [8], [9], [10] by bending or tubulating membranes and coalescing microdomains, e.g. in the case of SV40 and the AB5 toxins [11], [12], [13], [14] such as Cholera toxin B (CtxB) [15], [16], [17], [18]. The Alzheimer's disease-causing Aβ peptide, like these toxins, may cluster at the membrane, seeded by gangliosides, into oligomers or aggregates of anywhere from a few molecules to larger protofibrils, or large pore-forming annuli [19], [20], [21], [22], [23], [24], [25], [26], [27]. Lipid interactions, especially with gangliosides, have also been reported to induce conversion of the Aβ peptide, from random coil to α-helix, or helix to β-sheet [20], [25], [28], which is thought to enhance oligomerization and/or aggregation and affect uptake [29], [30].

The effect of such aggregation and clustering on the diffusion dynamics and stability of sphingolipid-binding ligands at the membrane has not been studied systematically. However, some evidence exists that higher-order oligomeric Aβ molecules or aggregates, rather than binding more tightly, lose the capacity to bind the membrane, and that primarily monomers or small clusters of the peptide associate with the membrane [29], [31], [32] and induce regions of immobility and/or decreased fluidity, depending on membrane composition [29], [33], [34]. It is not established whether unstructured aggregation would lead to membrane coalescence or stimulate uptake as does CtxB, which has a much greater affinity for the membrane than Aβ [25], [35], [36], [37]. In neither case is it known how aggregation affects mobility at the membrane.

The Sphingolipid Binding Domain peptide (SBD) is a fluorescently tagged peptide probe derived from the first 25 amino acids of the Aβ sequence, which we previously characterized as a tracer of sphingolipid trafficking pathways in the cell [38], [39] and of membrane dynamics of sphingolipid-containing domains [40], [41]. Identified by Fantini and his group as a potential glycosphingolipid (GSL)- and sphingomyelin (SM)-interacting sequence in Aβ, the isolated SBD peptide was previously shown by liposome capture experiments and surface plasmon resonance (SPR) assays [38] to interact with gangliosides. Somewhat surprisingly, this short sequence tagged with tetramethylrhodamine (TMR) showed a preference similar to what was reported for the full length Aβ1–40 peptide [25], [27], preferring more highly sialylated species GT1b and GD1a to GM1 at neutral pH, suggesting that a critical sphingolipid-targeting motif may indeed reside in these first 25 amino acids. SBD's interaction with the plasma membrane of cells was dependent on the presence of cholesterol, SM, and glycolipid [38], [39], [40], [41] similarly to full-length Aβ 1–40 and 1–42, which interact with phospholipids, cholesterol, and gangliosides [20], [23], [28], [37], [42], [43], [44], [45], [46], [47]. A characteristic cholesterol-dependent bimodal diffusion behavior of SBD at the membrane was furthermore demonstrated, like other ganglioside interacting probes such as CtxB [40], [41].

In the present study, using surface Plasmon resonance (SPR) and molecular dynamics (MD) simulations, we showed that variations in the linker construction and charge of the SBD peptide influenced binding strength to simplified, ostensibly plasma membrane-like ganglioside-containing membranes without inducing major structural changes. This gave us an interesting opportunity to test the effects of binding strength to gangliosides on diffusion behavior of the probes. As an indicator of the diffusion behavior of the peptides in the presence of the plasma membrane, we measured the different SBD variants by confocal fluorescence correlation spectroscopy (FCS) on live cell membranes. A comparison of the SPR binding and FCS assays revealed that an increase in aggregation propensity of certain SBD variants occurred at the plasma membrane, accompanied by very slow diffusion, and rather than improving binding, these aggregating variants showed poorer binding to ganglioside-containing mixtures.

## Materials and Methods

### Peptide preparation and cell labelling

**Figure 1:**
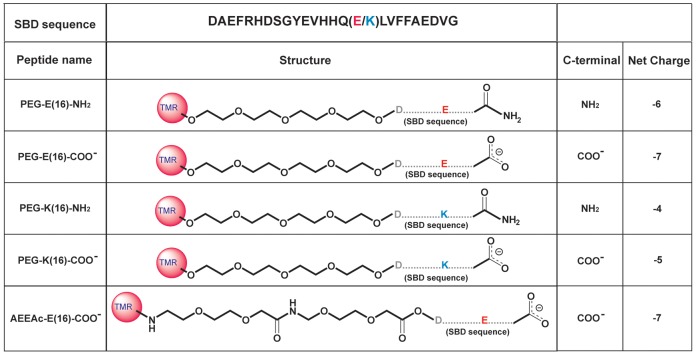
Structures of the five main SBD peptide variants used in this study. The linker structure between the peptide and the N-terminal TMR fluorescent tag is shown, as well as the C-terminal ending.

The SBD E16 variant with amino-ethoxy-ethoxy-acetyl (AEEAc)2 linker (fig. 1) was synthesised by Bachem (Switzerland), or by an in-house peptide synthesis core facility by standard Fmoc chemistry. For the polyethylene glycol (PEG)-linked variants of SBD, four PEGs were coupled to the N-terminal of the peptide as a spacer between the TMR dye and the peptide (fig. 1). PEG- and AEEAc-linked peptides had molecular masses of 3635 kDa and 3636 kDa, respectively. Handling and labelling of cells with SBD was done as described in [38], [41]. Cells labeled with peptides were imaged on a DeltaVision widefield fluorescence deconvolution microscope (Applied Precision, Inc., WA, USA) with a PLAPON 60×/1.42 NA oil-immersion objective from Olympus, and TRITC Semrock filters (New York, USA).

### SPR assays

Palmitoyl-oleoyl-phosphatidylcholine (POPC)/sphingomyelin (SM)/Cholesterol (Chol) (45∶25∶30 molar ratio), POPC/SM/Chol/GT1b (45∶25∶30 with 10% GT1b), POPC/SM/Chol/GD1a (45∶25∶30 with 15% GD1a), POPC/Chol (70∶30), or POPC/Chol/GT1b (70∶30 with 10% GT1b) liposomes were prepared in HBS-N buffer (10 mM HEPES, 150 mM NaCl) pH 7.4 using a standard extrusion method. Briefly, liposomes were prepared by mixing the different components together in a glass vial at room temperature, and dried under a nitrogen stream. To remove the organic solvent in which the components were dissolved, the lipid was lyophilized for at least two hours. The final lipid concentration of 0.5 mM was reached by re-dissolving the lipid film in 1 mL HBS-N and vortexing thoroughly for 10 min. The formation of a lamellar structure was supported by freezing the mixture in liquid nitrogen, followed by thawing in a hot water bath, repeated five times. The lipid film was completely removed from the inside wall of the glass vial by bath sonication in ice water for at least 30 minutes. The solution was extruded through a 50 nm membrane 51 times to yield small lipid vesicles. The liposomes produced were stored at 4°C for no longer than one week between experiments to prevent the formation of liposome clusters. The size of the liposomes was assessed by Dynamic Light Scattering on a Malvern Zetasizer (Worcestershire, UK).

Lipids were from Avanti (Alabaster, AL) or Carbosynth (Compton, UK). Solvents and other reagents were from Fisher Scientific (Waltham, MA), Merck (Whitehouse Station, NJ), GE Healthcare (Uppsala, Sweden), and Calbiochem (San Diego, CA). SPR assays were carried out on a Biacore 3000 instrument with L1 chips (GE Healthcare), according to [41]. The tubing was cleaned prior to each experiment according to the method given by the manufacturer with 40 mM OG and de-ionized water and left on standby overnight in Milli-Q water. Liposome solutions were injected until the immobilization level reached at least 5000 RU, except for POPC/SM/cholesterol/GD1a and POPC/cholesterol/GT1b mixtures, for which only lower immobilizations could be reached.

Peptide solutions were prepared freshly in HBS-N buffer, pH 7.4 (see above) to give a final concentration of 35 µM. 40 µl of peptide was injected over the lipid surface at a flow rate of 10 µl/min and the peptide-surface complex was allowed to dissociate in buffer for 15 min. The carrier buffer HBS-N was injected prior to the peptide injection and each peptide injection was performed on a freshly prepared surface.

**Figure 2:**
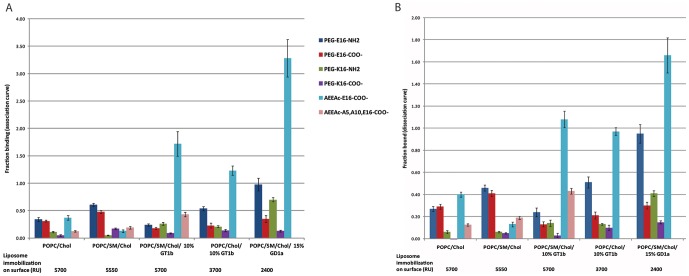
Surface Plasmon Resonance (Biacore) assay of SBD variants' association with immobilized liposomes. Surfaces with and without sphingolipid and ganglioside consisted of the mixtures listed below the X-axis. (A) Fraction binding on the Y-axis refers to the peak height of the association curve in response units (RU), normalized to the average immobilization level over all flow cells for that experiment, indicated by the numbers (in RU) under the graph. The value for fraction binding was obtained by averaging this normalized peak value for at least three flow cells (see Materials and Methods). (B) Fraction bound on the Y-axis refers to the height of the dissociation curve in RU, normalized to the average over all flow cells for that experiment. The error bars represent the variation in the magnitude of response between flow cells.

The values given in the graph in fig. 2 were an average from at least 3 flow cells. The “fraction binding” value in fig. 2A was taken from the association phase data, excluding the first 30 seconds after the start of peptide injection. Fraction binding was calculated by taking the difference between the height of the curve in response units (RU) at the start of the peptide injection phase from the height of the curve at the end of the peptide injection phase (see fig. S1), divided by the normalized immobilization level. The normalized immobilization level for a flow cell was the amount of liposome bound to the chip at that flow cell, after the value at each flow cell had been normalized to the average over all flow cells. The value for “fraction bound” in fig. 2B was determined by taking the height of the curve (in RU) after 15 mins of dissociation, divided by the normalized immobilization level.

### FCS experiments

FCS experiments were done on an Olympus FluoView 300 confocal microscope with the custom-built FCS detection unit on top of the scanning unit. A 543 nm helium-neon laser was used to excite the TMR fluorophores and the laser power was maintained at 25 µW for all experiments. Details of the instrumentation are described in [41]. The fluorescence signals were collected onto a single photon sensitive avalanche photo diode (APD; SPCM-AQR-14, Pacer Components, UK) after passing through an emission filter 595 AF60 (Omega Optical Inc., Brattleboro, VT) in point-scanning mode, and fitting of autocorrelation curves was done as described [41] using a self-written program in IgorPro 6.0 (Wavemetrics, Portland, OR) with 2D or 3D and 1- or 2-particle, 1 triplet models, yielding diffusion times τ_D1_ and τ_D2_ and corresponding mole-fractions F1 and F2.

2D2P1t:
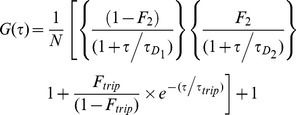



3D1P1t:
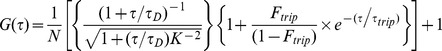
Here *N* refers to the number of particles in the observation volume; *τ_D1_* and *τ_D2_* are the characteristic diffusion times of different particles; the mole fraction of particle 2 is given by *F_2_ = 1-F_1_*; the fraction of molecules in the triplet state and the characteristic triplet state time are given by *F_trip_* and *τ_trip_*, respectively. It should be noted that the mole fractions *F_1_* and *F_2_* are strongly dependent on the actual molecular brightness of the molecules with brighter molecules contributing more strongly to the autocorrelation function and biasing the fractions towards the brighter species. In our experiments, where we have possible aggregates of fluorescent peptides, one would expect an overestimated fraction of the aggregates.

In a homogeneous solution of fluorescently labelled probes, the autocorrelation function will be reproducible over many measurements with little deviation. However, in heterogeneous solutions in which there are rare components with a higher brightness and a lower diffusion coefficient, one will detect characteristic intensity peaks or spikes of the brighter and rarer species and a change of the characteristic shape of the correlation function to a model of higher complexity (two or more particles instead of a single diffusive component) in a subset of the measurements.

Quantification of the occurrence of spikes in the fluorescence intensity traces and deviation from normal fitting in the autocorrelation curves, respectively, was done manually according to the following criteria: a spike was defined as a sudden jump in the intensity trace of more than five times the fluctuation range (highest to lowest) around the average intensity for that sample. Autocorrelation curves that could not be fitted in the longer time ranges (i.e. >1 sec lag times) to the normal 2-particle fitting model were assessed by visual inspection; abnormally long diffusion times, indicative of immobility, together with spikes in intensity, which correlated strongly with distortions in autocorrelation curves at long time ranges, were taken as indicators of probe aggregation.

### REMD simulations

200 nanosecond (ns) replica-exchange molecular dynamic (REMD) simulations [48] for every SBD monomer were performed using the Gromacs 4.5 program [49], [50], [51]. 32 replicas were applied for each REMD simulation at 297 to 554°K. The final overall exchange rate was larger than 50%. Amber 99SB force-field parameters [52] were applied in this REMD study. Each monomer model was solvated in implicit solvent with no pressure [53]. The time-step was set to 2 fs throughout the simulations. V-rescale coupling [54], [55] was used for the temperature control. No pressure coupling was used in this study. The pair-list of the non-bonded interaction was recalculated every 10 time-steps with a pair-list cut-off distance of 10 Å. The LINCS routine [56] with a tolerance of 10E-4 was used in all simulations. The atomic coordinates and velocities were saved every 5 ps. The secondary structure of the protein was assigned by the DSSP program [57]. The secondary structure probability and backbone dihedral angle distribution were analyzed in Ramachandran plots for each residue (fig. S2).

### Circular Dichroism (CD) Measurements

Circular dichroism measurements were performed on a Chirascan spectropolarimeter (Applied Photosystems) using a rectangular 1 mm quartz cell at 23°C. Data were acquired with a pitch of 1 nm between 270–190 nm with an averaging time of 1 sec. 10 scans were averaged and smoothened for each sample. Peptide concentrations in 50 mM potassium phosphate buffer, pH 7.4, were determined by measuring the absorbance of the dye (TMR) coupled to the peptide, using a Cary 300 Bio UV-Vis Spectrometer (Varian) at 556 nm. The extinction coefficient for TMR was 90000. Ultimately, all spectra were baseline corrected and converted into mean residue ellipticities (MREs) using Eq. 1:
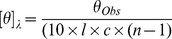
(1)where [θ]λ is the MRE at wavelength λ in deg cm^2^ dmol^−1^, l is the path length in cm, c is the concentration in M, *n* is the number of residues and (n-1) designates the number of peptide bonds in the studied polymer. In 50% trifluoroethanol (TFE) the AEEAc-linked peptide changed conformation into predominantly helical, indicating that the presence of linker does not obstruct the peptide from undergoing conformational changes.

## Results

### 1. Charge interactions are not critical to ganglioside binding of a sphingolipid-interacting peptide probe, the SBD

A fluorescently tagged ganglioside- and sphingomyelin-interacting 25 residue peptide derived from the amyloid peptide Aβ, the Sphingolipid Binding Domain (SBD) probe (shown in fig. 1) was described in previous work as a tracer of sphingolipid-containing domains in cellular membranes. SBD binds in the micromolar range to a constellation of GSL, SM, and cholesterol, with GT1b being the optimum ganglioside among several tested by liposome-capture studies and SPR [38].

Since SBD does not possess the toxic properties of Aβ [38] and lacks the most aggregation-prone, β-sheet forming region of Aβ between amino acids 28 and 42 [20], [22], [58], it allows us to examine the consequences of glycosphingolipid (GSL) binding on diffusion behavior, independent of fibrillization, pore-forming, or penetration effects. The binding preference of the −7 net charged SBD peptide AEEAc-E16-COO- is influenced by pH, with lower pH buffers giving much stronger interactions with less-sialylated gangliosides, notably GM1 [38], [47], [59]. It has not been tested whether a possible repulsive effect of the sialic acid groups with the negatively charged peptide might be overcome by reducing the net negative charge. We tested this idea by introducing stepwise increases in the overall charge of the peptide and monitoring their effect on binding by SPR measurements on immobilized POPC/SM/Chol (45∶25∶30 molar ratio) or POPC/Chol (70∶30) liposomes [60] with 10% or 15% GT1b or GD1a ganglioside, on dextran-L1 chips (GE Healthcare). All SPR binding curves obtained with the 5 peptide variants (whose compositions are shown in fig. 1) on different lipid mixtures are given in figure S1, and the quantification of binding at the peak of the association curve, and after dissociation are shown in figure 2A and B (“fraction binding” vs. “fraction bound”; see Methods for explanation of how the values were obtained).

A naturally occurring variant of Aβ with glutamate at position 16 instead of lysine does not lead to major structural changes, according to an NMR study of Aβ1–28 [61]. This gave us the opportunity to avoid inducing drastic structural changes in the peptide, while increasing the net charge, by introducing K16 in place of E16 in the original SBD sequence (fig. 1). The fraction of peptide bound at the peak of the association curve (fig. 2A), and the fraction remaining bound after dissociation (fig. 2B) were, surprisingly, consistently lower on GSL for the PEG-K16-SBD peptides than the equivalent E16 SBD (purple vs. maroon; olive vs. dark blue). For the non-GSL containing surfaces, the K16 peptides bound even more poorly in comparison to all three E16-containing peptides (olive/purple vs. dark blue/maroon).

When the C-terminal carboxylic acid was modified by an amide (NH_2_), increasing the positive charge by an additional +1 (see fig. 1), the K16 and E16 peptides showed improved binding to GSL (olive vs. purple; dark blue vs. maroon, fig. 2), in particular when more ganglioside was present, in the POPC/SM/Chol/15% GD1a mixture. However, it is notable that these less negative peptides did not perform nearly as well as the most negative AEEAc-E16-COO- peptide (light blue), suggesting that simply increasing the positive charge of the probe does not improve binding to acidic gangliosides per se. Rather, it appears that the presence of an amide group, either as an addition at the C-terminus, or via the AEEAc linker, improves binding strength to ganglioside containing mixtures, especially in the context of the E16 peptide.

### 2. An amide-containing PEG linker stabilizes binding to ganglioside-containing surfaces

Alternate versions of the peptide were synthesized, with AEEAc2 replaced by (PEG)4 (fig. 1), to examine the effect of removing the amine group within the linker. The AEEAc2- peptide (light blue bars in fig. 2) exhibited higher binding than the equivalent PEG-peptide (maroon), as well as the more positively charged PEG peptides (dark blue, olive, purple), as long as GSL was present (POPC/SM/Chol/10% GT1b or 15% GD1a), but this was not exclusively due to the AEEAc linker, since a sequence mutated at two amino acids (R5A, Y10A) that binds much less well to cell membranes [38] showed reduced binding (pink).

Inclusion of SM in the GSL mixture improved binding of AEEAc-E16-COO- slightly (light blue, fig. 2), but did not have a positive effect on the other peptides (dark blue, maroon, purple). In contrast, when SM was present, but no ganglioside, in the POPC/SM/Chol mixture, the PEG-E16 peptides performed better than the AEEAc-E16-COO- (dark blue and maroon vs. light blue). These results indicate a positive interaction between SBD and SM, that also depends on the ganglioside surroundings, and that may be influenced by the presence of an amine in the linker. The linker-borne amine, however (light blue), does not seem to have the equivalent effect to a C-terminal amine in an otherwise similar peptide (dark blue).

To differentiate between electrostatic effects and specificity for a particular ganglioside, we substituted 15% GD1a, which carries two negative charges, for 10% GT1b, which has three, in an otherwise identical lipid mixture. All of the SBD peptides showed improved binding to the higher percentage GD1a-containing mixture (fig. 2). GD1a is much more highly represented than GT1b in the SH-SY5Y neuroblastoma cells used in the FCS experiments [62] (see sections 4–6, below), but was found to be slightly less favored than GT1b in previous liposome capture experiments with SBD [38]. In summary, inclusion of an amine-containing linker or terminus, but not changes in overall charge per se, lead to substantial improvements in the ability of the peptide to bind to plasma membrane-like GSL-containing surfaces.

### 3. Replica exchange simulations show no change in relative amounts of structural motifs, but an increased frequency of random coil in the best binding SBD variants

**Figure 3:**
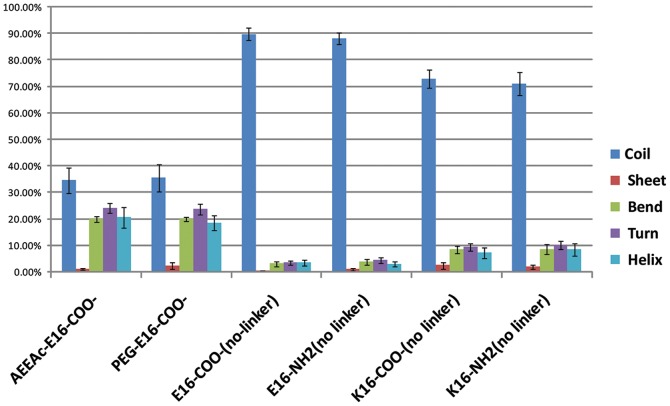
Secondary structure probability distributions for the different SBD variants. Secondary structure propensity (DSSP) analysis from 200 ns REMD simulations, showing the percentage of the time that each structure occurs. Amino-PEG (AEEAc2)-linked and PEG4-linked forms of the E16 peptide with a carboxy terminus were compared in order to assess possible effects of the linker on structure. The different linkers appeared to have no significant effect on the occurrence of structural features. The last four groups show the distribution of structural features that occur in the four variants, either E16 or K16, and with carboxy (COO-) or amide (NH_2_) termini, in the absence of linker. The inclusion of E vs. K at position 16 affects the frequency of random coil, but does not appear to otherwise change the relative amounts of structural features. Ramachandran plots showing the distribution of peptide bond angles, and exact frequencies of each structural feature are given in fig. S2.

We modeled the different SBD monomer variants by replica-exchange molecular dynamics (REMD) simulations, which enhance the conformational sampling of peptides by combining high temperature simulations with a Monte Carlo algorithm. The generally poorer binding of the K16 containing peptides was not explained by major structural changes, since relative amounts of β-sheet, turn, bend, and α-helix were similar, and in the absence of linker only marginally increased β-sheet content. In simulations of E16-SBD with AEEAc or PEG4 linker, the conformation of the peptide stabilized from ∼90% random coil to being more structured (fig. 3). Ramachandran plots and numerical frequencies of the occurrence of each structural feature are shown in fig. S2. No significant differences in the frequency of the structure classes were noted between the AEEAc and PEG linker, although β-sheet was slightly but not significantly increased in the PEG-linked version at the apparent expense of α-helix (fig. 3; S2). The K16E change did not significantly alter the frequency of different structural features.

In concurrence with the REMD simulations, CD measurements of peptides in 50 mM potassium phosphate buffer, pH 7.4, did not reveal any detectable differences in structure, and showed the conformation of all the peptides to be random coil (not shown).

### 4. SBD variants that compromise ganglioside binding diffuse slowly

**Figure 4:**
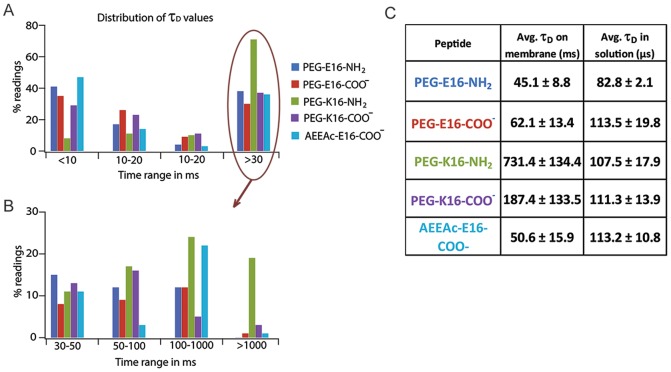
Histogram and average diffusion times derived from FCS measurements on SBD variants in neuroblastoma cells. (A) Histogram distribution of τ_D_ values with the confocal volume centered on the plasma membrane, over the indicated times ranges in milliseconds for all five SBD variants listed in the key. All variants show a bimodal distribution of τ_D_ values except for weakest binding PEG-K16-NH2. The >30 ms group in (A) is redistributed in (B) over the indicated time ranges. (C) Table of average τ_D_ values on the membrane and in solution ± standard deviation. Average values were derived from n>60 measurements for each probe either at the membrane or in solution.

We asked whether variants that compromised binding to GSL would have consequences for diffusion behavior on actual cell membranes. Confocal FCS measurements were carried out on neuroblastoma SH-SY5Y to measure diffusion times, τ_D_, defined as the average transit time through the confocal volume. For FCS measurements on the membrane, autocorrelation functions (ACFs) fitted a 2D, 2P model, indicative of the probe falling into two diffusion classes deriving from bound and unbound molecules. As expected [41] a bimodal distribution was seen for all the SBD variants, with the exception of PEG-K16-NH2 (fig. 4). Notably, the two best ganglioside-binding SBD variants, AEEAc-E16-COO- and PEG-E16-NH2, displayed very similar bimodal distribution profiles and diffusion times of ∼50 ms (fig. 4A–C). The worst-performing peptides with respect to ganglioside binding–particularly PEG-K16-NH2–were also the slowest-diffusing, having average τ_D_ values much longer than the better-binding E16 versions (fig. 4C).

### 5. SBD variants that compromise ganglioside binding aggregate at the cell membrane

**Figure 5:**
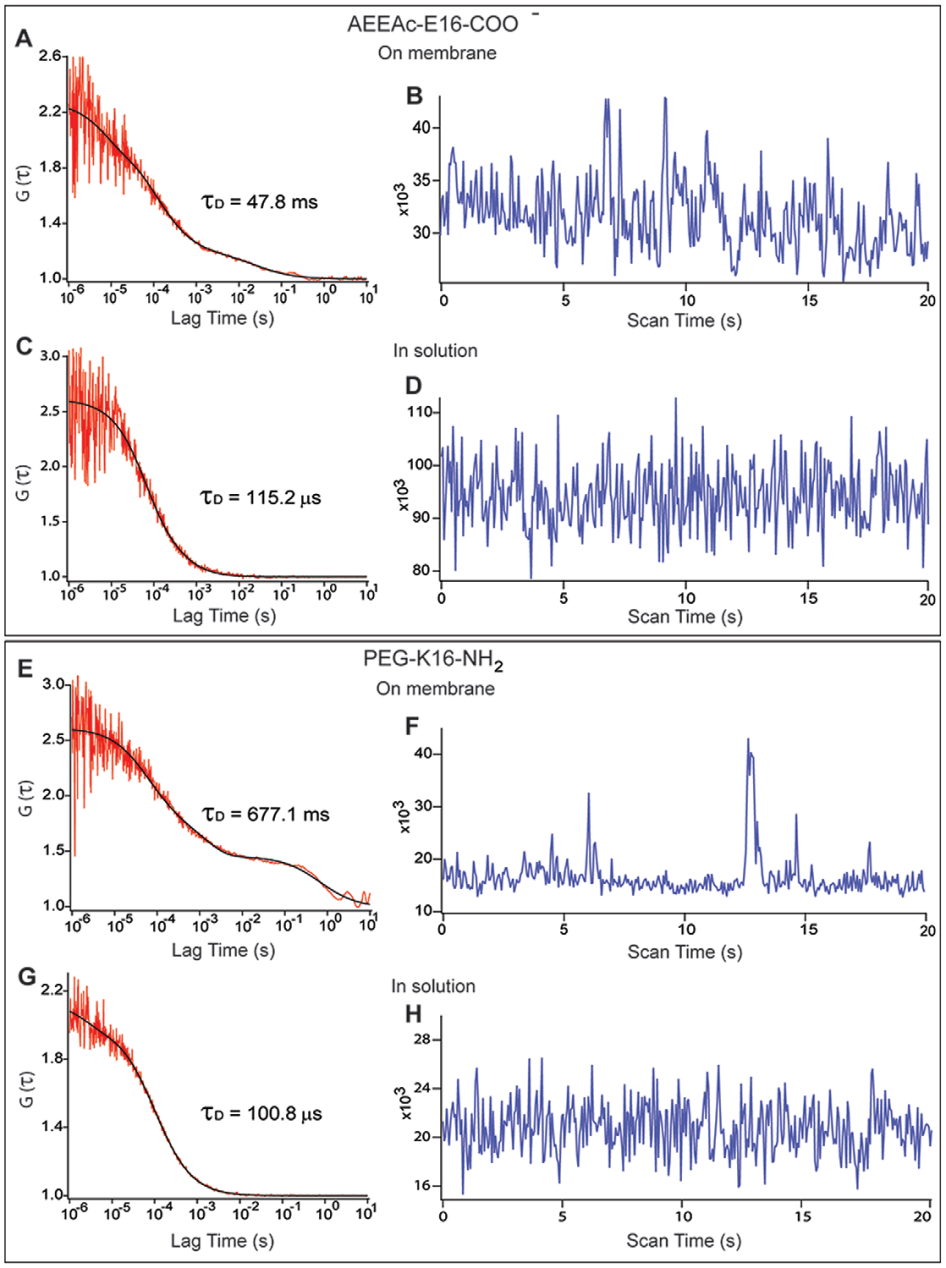
FCS autocorrelation curves and intensity traces on SBD variants labelling neuroblastoma cells. Normalized ACFs (left; traces) and 2D,2p models (left; line curves), with lag time of the autocorrelation function G(τ) indicated in seconds on the X-axis. Corresponding intensity traces are given on the cell membrane (A, B, E, F) and in the solution (C, D, G, H) for SBD with either the amine-PEG AEEAc linker, E16, and COO- terminus (A–D), or for SBD with a PEG linker, K16, and an amidated C-terminus (E–H). Deviations in the ACF from the fitting model occur between ∼1–10 sec in the measurement of PEG-K16-NH2 on the membrane (E), and spikes in the corresponding intensity fluctuation trace for the membrane measurement in (F) are evident, but not in the ACFs and intensity traces for AEEAc-E16-COO- (A, B) or for either peptide in solution (C, D, G, H). τ_D_ values indicated correspond to the particular graphs shown in the figure and not the average values.

**Figure 6:**
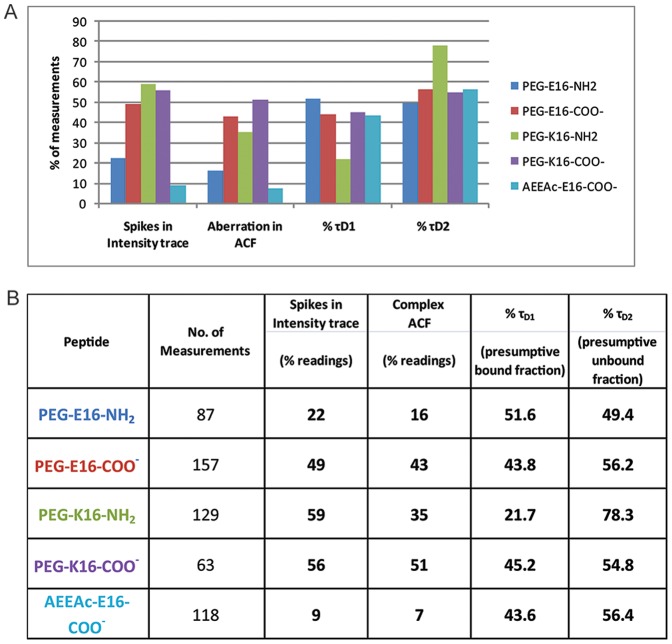
Frequency of aggregation and non-binding of SBD variants. Spiked intensity traces and complex ACFs by FCS indicated aggregation of SBDs. A complex ACF is defined as deviating from the fitting model in the time-lag range of 1–10 sec. The last two columns show the fraction of peptide assumed to be bound to the membrane, based on %τ_D1_ (millisecond range) vs. %τ_D2_ (microsecond range). Spikes were counted in each measurement over 20 seconds.

In the SPR assays, peptides with the amide AEEAc-linker bound better to ganglioside-containing surfaces than PEG-only linker, and the PEG-E16 with amidated C-terminus was in turn superior to those with K16 or without the amidation. Binding efficacy was also associated with particular FCS diffusion profiles on cell membranes. Typical examples of intensity traces and ACFs of the different SBD variants applied are shown for AEEAc-E16-COO- and PEG-K16-NH2, the best- and worst-binding variants, respectively (fig. 5A–D, E–H). There was a high frequency (22–59%; fig. 6) of aggregation in PEG-linked peptides, indicated by fluorescence intensity spikes, like those in fig. 5F. The occurrence of spikes was much lower for the AEEAc2-containing SBD (9%; fig. 5B; summarized in fig. 6). Intensity spikes were notably absent from measurements of any of the peptides in solution. As with the diffusion times, the two SBDs that interacted most strongly with GT1b by SPR (AEEAc-E16-COO- and PEG-E16-NH2), were also the two that had the fewest intensity spikes (fig. 6).

Measurement of the fraction of particles with longer diffusion times, τ_D1_, vs. shorter diffusion times, τ_D2_, gives information about the total fraction of probe bound, because freely diffusing unbound probe gives exclusively short τ_D_s, in the range of hundreds of µsec, while membrane-bound probes typically have τ_D_s of ∼1–100 msec [39]. The poorly binding PEG-K16-NH2 peptide not only had the greatest frequency of intensity spikes, but also showed an unusually low fraction (21.7%) of bound τ_D1_ particles, vs. 43.8–51.6% for the other variants (fig. 6B).

In some cases, ACFs deviated from the 2D, 2P model in the 1–10 s range of the curve (e.g. fig. 5E). These complex correlation functions were seen predominantly for the poorly GSL-binding PEG-linked SBDs, and presumably arose from a small fraction of particles having extremely long diffusion times (see above, and fig. 4B). Intensity spikes arising from large fluorescent particles diffusing through the confocal volume were strongly correlated with complex ACFs like those obtained with PEG-K16-NH2 (figs. 5E; see [63]). Immobile aggregates of the K16 peptides at the membrane may produce long diffusion times (>100 ms; fig. 4C), and bleach, distorting the ACFs [64], [65], [66].

**Figure 7:**
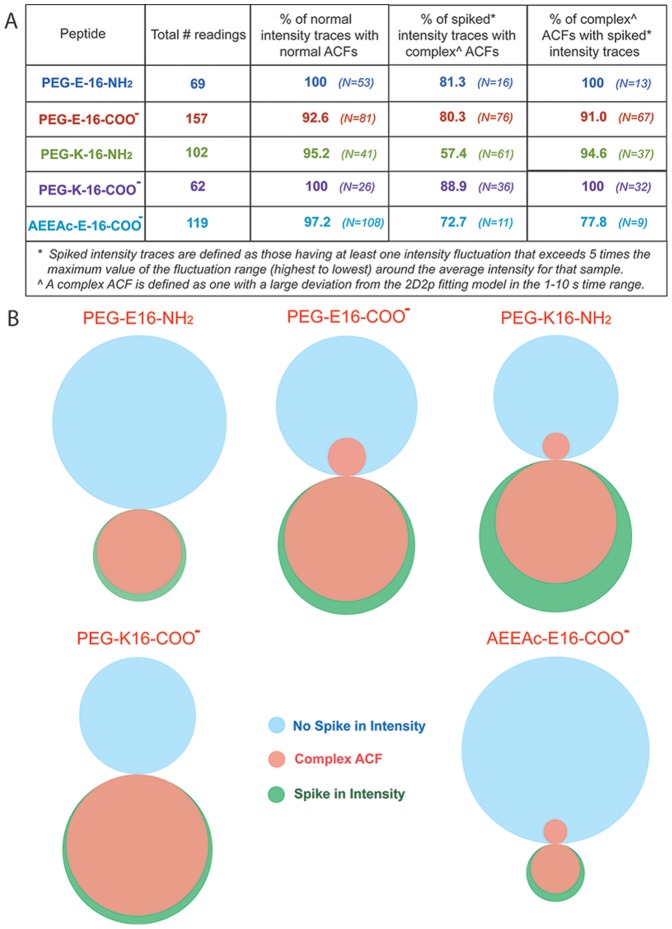
Correlation of aggregation-induced intensity spikes with complex autocorrelation curves in the different SBD variants. (A) Correspondence of spikes in intensity traces with incidence of non-fitting of the ACF with the 2D,2p model (curves in fig. 4) between 1–10 sec (“complex” ACFs). The number of ACFs and intensity traces observed in each case is given in brackets. For the AEEAc-E16-COO- peptide, the number of spiked intensity traces and complex ACFs was very low compared to the others (n = 9 and 7, respectively). (B) Venn diagrams illustrating the degree of correspondence between readings showing spikes in intensity traces (green circles), vs. those that do not (blue circles) and readings having complex ACFs (beige circles), for each SBD variant. The actual relative proportions of readings in each category, as well as the proportion of readings that were shared between categories, are represented by the relative sizes of the circles and overlaps.

The frequency of complex ACFs with non-fitting in the 1–10 s range is given in fig. 6, and their correlation with spikes in intensity traces is shown as a Venn diagram in fig. 7. This diagram shows schematically the degree of correspondence between complex ACFs and the occurrence of spikes in intensity traces, with the size of the overlapping areas proportional to the amount of actual overlap between groups. In general, spiked intensity traces were also those that resulted in complex ACFs displaying distortions at longer lag times, e.g. for PEG-E16-NH2 and PEG-K16-COO- peptides, these were completely overlapping. However, it was noted that for the worst-binding PEG-K16-NH2 peptide, the correlation between spiking and complex ACFs is less good than for the others (i.e. the overlap between beige and green is less complete), suggesting that in this case, aggregated slow particles dominated the ACF, rather than being a rare occurrence that only distorted it at long lag times (see fig. 7). Non-aggregating membrane dyes such as 1,1′-Dioctadecyl-3,3,3′,3′-Tetramethylindocarbocyanine Perchlorate (DiI) do not produce complex ACFs [40], [41]. The fact that intensity spikes and complex ACFs appeared only in readings at the plasma membrane, and were negligible for all peptides in solution, suggests that aggregation of any of the SBD variants occurred exclusively upon contact with the cell membrane.

### 6. All SBD variants are endocytosed by cells

**Figure 8:**
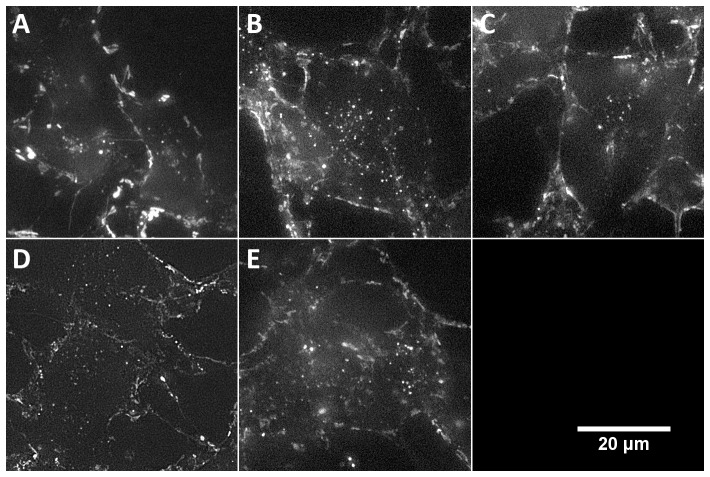
Labelling of neuroblastoma SH-SY5Y with SBD variants. SBD was incubated with neuroblastoma SH-SY5Y for 30 mins at room temperature, washed, and imaged by widefield fluorescence microscopy after 1 hour. Five mid-cell optical sections were projected after deconvolution. All peptides can be seen to have been endocytosed into vesicular compartments. Peptides were: A) PEG-K16-NH3; B) PEG-K16-COO-; C) PEG-E16-NH2; D) PEG-E16-COO-; E) AEEAc-E16-COO-. Scale bar is 20 µm.

Cell labeling on neuroblastoma SH-SY5Y as was done for the FCS experiments, and subsequent wide-field fluorescence imaging after 1 hour of uptake, showed that all variants of the peptide were endocytosed by the cells, with no obvious difference in surface distribution (fig. 8), even though diffusion and aggregation behavior clearly differed between peptides. Any differences in surface distribution may be undetectable by conventional fluorescence microscopy due to a resolution limit likely exceeding the dimensions of the range of areas bound by the peptide at the membrane.

## Discussion

In this study, we compared the binding and diffusion behaviour of several variants of the Aβ-derived SBD (Sphingolipid Binding Domain) probe that consisted of a change in the charge and terminal modification of the peptide, without an overall change in structure. The variations changed the ability of the SBD to bind to its usual targets—gangliosides GT1b and GD1a in plasma membrane-like mixtures, and this decrease in binding propensity to GSL correlated with an increase in aggregation behaviour at the plasma membrane, and slowed diffusion by FCS.

Variants of the SBD containing a positive K16 in place of negative E16, although as expected [61] did not give any structural changes in MD simulations, led to relatively large changes in interactions with gangliosides. Surprisingly, the two K16 variants displayed the weakest interactions with SBD's target GSL, GT1b [38]. These results indicate that interaction between the SBD peptide and ganglioside-containing surfaces is not non-specifically electrostatic in nature, and may depend upon other interactions, e.g. CH-π stacking typical for lectin binding to carbohydrate [3]. This was also proposed to have a role in the binding of glycolipid sugar-rings to aromatic residues in Aβ [47], [67] as well as the sphingolipid binding domains of various proteins.

Most SBD variants showed one slow population with τ_D_ ∼50 ms or longer, which was earlier shown to be cholesterol-dependent [41], and a second faster-diffusing non-cholesterol-dependent population, having τ_D_ ∼1–10 ms. In contrast, the poorly binding PEG-K16-NH2 variant showed a much longer (i.e. slower), unimodal τ_D_ distribution than the others, with PEG-K16-COO- being a distant second, indicating a lower mobility of this variant. PEG-K16-NH2 also had correspondingly the highest number of intensity spikes and lowest bound fraction to cell membranes, whereas the τ_D_ values of all the variants were similar in the extracellular solution. Measurements in solution produced intensity traces that lacked spikes and fitted well with 3D,1P diffusion models, suggesting that any aggregation that did occur happened only after contact with the plasma membrane. Notably, inclusion of the AEEAc linker appeared to suppress aggregation, at the same time that it improved binding capacity to GSL in the SPR studies.

These observations are consistent with ganglioside-seeding models of full length Aβ's interaction with the membrane [68], and suggest that the first 25 aa may be sufficient for this seeding activity, as described by Williamson et al [69]. Moreover, the fact that the more common K16 isoform [61] exhibits more membrane-induced aggregation and less avid membrane binding in our shorter SBD peptide, suggests a possible preference of the E16 mutation for binding in monomeric or small oligomeric form, because of ganglioside interactions. While it is surprising that the more positively charged K16 and C-terminal NH_2_ versions bind less well to negative gangliosides in our hands, this is also consistent with the findings of Ikeda et al [47] and others who postulate that hydrogen bonding and CH-π interactions of aromatic amino acids in Aβ with sugar head-groups of gangliosides are more important determinants of the interaction, and may in fact protect against aggregation [70]; indeed this would be one plausible explanation for the results we see, where peptides that bind better to gangliosides actually aggregate less. For the weaker ganglioside binders, one can imagine that the peptide might not aggregate in solution where individual peptide molecules are freely diffusing in three dimensions, but when loosely bound to the membrane by a weak lipid interaction, they could come into close enough proximity in only two dimensions, such that aggregation occurs more readily, and is not hindered by interaction with gangliosides. An alternative model derived from FRET studies of fluorescent Aβ with lipid vesicles states that GM1 does not bind to Aβ with appreciable affinity, but rather serves as a seed for the fibrillization that occurs upon contact with the membrane, switching Aβ's affinity to POPC [71]. This may also be consistent with our findings.

Membrane-induced aggregation may be similar to the prion protein (PrP) which is normally found in non-aggregating α-helical form (PrP^C^), but upon interaction with membranes can be induced to form β-sheet and convert to the pathogenic PrP^Sc^ form [72], [73]. Critchley et al [73] found in SPR experiments that while both bound to membranes, the oligomerizing β-sheet form PrP^Sc^ bound more strongly than the α-helical form, in contrast to our experience with SBD. Similar findings were reported by Inaba et al for Aβ full-length peptide [74]. However, the α-helical form of Prp also aggregated at high concentrations, particularly in the presence of negatively charged membranes [73]. The binding was stronger at low pH for both forms. This was attributed to protonation of histidines in PrP, and consequently stronger electrostatic interactions with the negative membrane.

Interestingly, this is reminiscent of the behaviour of Aβ, which also binds more strongly at low pH, and contains two histidines at positions 13 and 14 of the peptide, at least one of which is involved in binding to the sialic acid of GM1 [69]. In that report, it was found that the more N-terminal region of Aβ, which is within the sequence that we used for the SBD (aa 13–17) is responsible for the interaction, and is induced upon interaction with the ganglioside to convert from unstructured to α-helix. Accordingly, (and consistent with [31]) binding to GM1 was unrelated to oligomerization in their findings. In our earlier characterization of SBD, we described a similar effect to these observations on Aβ, where low pH not only strengthened binding considerably in liposome capture experiments, but it changed the ganglioside preference from more negative polysialylated gangliosides like GT1b and GD1a to mono-sialylated GM1 [38](see [59]).

Our results that more aggregation-prone species of the peptide interact less well with the membrane are perhaps surprising in light of the fact that toxins such as CtxB bind more tightly upon oligomerization, as one would expect, and that clustering of ganglioside targets by toxins and viruses indeed seems to be part of the infective strategy of these agents by fostering uptake into endocytic domains [1], [7], [9]. Additionally, Aβ monomers have been reported to bind with less overall affinity than aggregates, although this comes about because of a difference in relative on- and off-rates between the two species [35], [74]. Even though the more aggregation-prone variants of SBD had an apparently lower affinity for ganglioside-containing surfaces by SPR, as well as lower bound fractions by FCS on cell membranes, their longer diffusion coefficients indicate that they were less mobile at the cell surface, once bound. This may be consistent with the findings of Kremer et al [75] who determined by fluorescence anisotropy that aggregated Aβ increased membrane order, and that this was enhanced by the presence of gangliosides. The results presented here also demonstrate that the β-sheet forming part of the Aβ peptide is not necessary to induce aggregation at the membrane.

## Conclusion

In this study, we observed a striking inverse correlation between the binding ability of SBD variants to ganglioside-containing membranes, and membrane-induced aggregation. Aggregation of the peptide, rather than increasing binding to cells, correlated with low binding and very slow diffusion behaviour. As yet, we cannot distinguish whether aggregation inhibits binding, or weak binding fosters aggregation–however, aggregation occurring only at the membrane implies the latter. This may suggest a mechanism whereby a stronger interaction of Aβ (or any membrane binding peptide) with ganglioside targets, in the absence of the formation of higher order structures, could favour low-valency binding vs. aggregation. More generally, the information from this study begins to tell us about correlations between the aggregation behaviour of a peptide, its interaction with particular lipids, and its membrane diffusion properties. Once enough data is accumulated describing the relationship between peptide features and their behaviours on membranes, it may be possible to construct peptides that are engineered toward particular interactions with the membrane.

## Supporting Information

Figure S1 **SPR curves showing association at ∼110 sec and dissociation at ∼350 sec of the different SBD variants, in response units (RU) on the Y-axis, and time (seconds) on the X-axis.** The compositions and molar ratios of the liposomes immobilized on the L1 chip are given in the titles of each graph. Red: PEG-K16-NH2; Green: PEG-K16-COO-; Blue: PEG-E16-NH2; Magenta: PEG-E16-COO-; Gray: AEEAc-E16-COO-.
(TIF)

Figure S2 **Ramachandran plots (A), with Phi (x-axis) and Psi (y-axis) showing the dihedral angles of the peptide backbone, which usually reflect the secondary structure; (B) secondary structure propensity (DSSP) analysis from 200 ns REMD simulations, with each number representing the fraction of the time that each structure occurs.** The effect on SBD peptide structure was first compared between the AEEAc and the PEG linker. The distribution of structural features appeared to be very similar between the two. In the absence of linker, the distribution of structural features was compared between the four variants, either E16 or K16, and with carboxy (COO-) or amide (NH_2_) termini, in order to assess possible effects of these modifications on the structure in isolation.
(TIF)
