# Supplementary figures and images for: Weak Glycolipid Binding of a Microdomain-Tracer Peptide Correlates with Aggregation and Slow Diffusion on Cell Membranes
Source: PLoS One. 2012 Dec 12;7(12):e51222. doi: 10.1371/journal.pone.0051222 (PMC3520979; doi:10.1371/journal.pone.0051222)

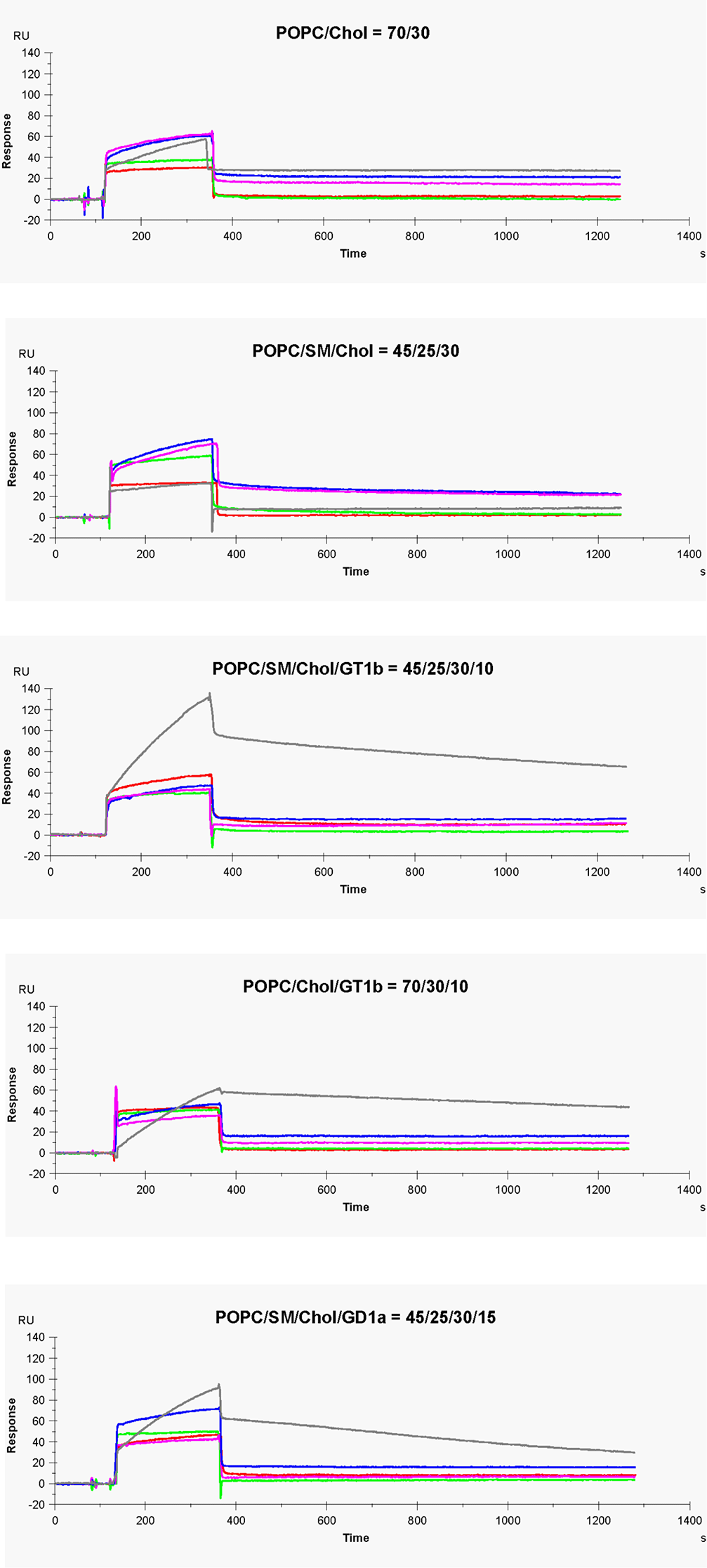

Supplement: Figure S1 — SPR curves showing association at ∼110 sec and dissociation at ∼350 sec of the different SBD variants, in response units (RU) on the Y-axis, and time (seconds) on the X-axis. The compositions and molar ratios of the liposomes immobilized on the L1 chip are given in the titles of each graph. Red: PEG-K16-NH2; Green: PEG-K16-COO-; Blue: PEG-E16-NH2; Magenta: PEG-E16-COO-; Gray: AEEAc-E16-COO-. (TIF) [file pone.0051222.s001.tif]

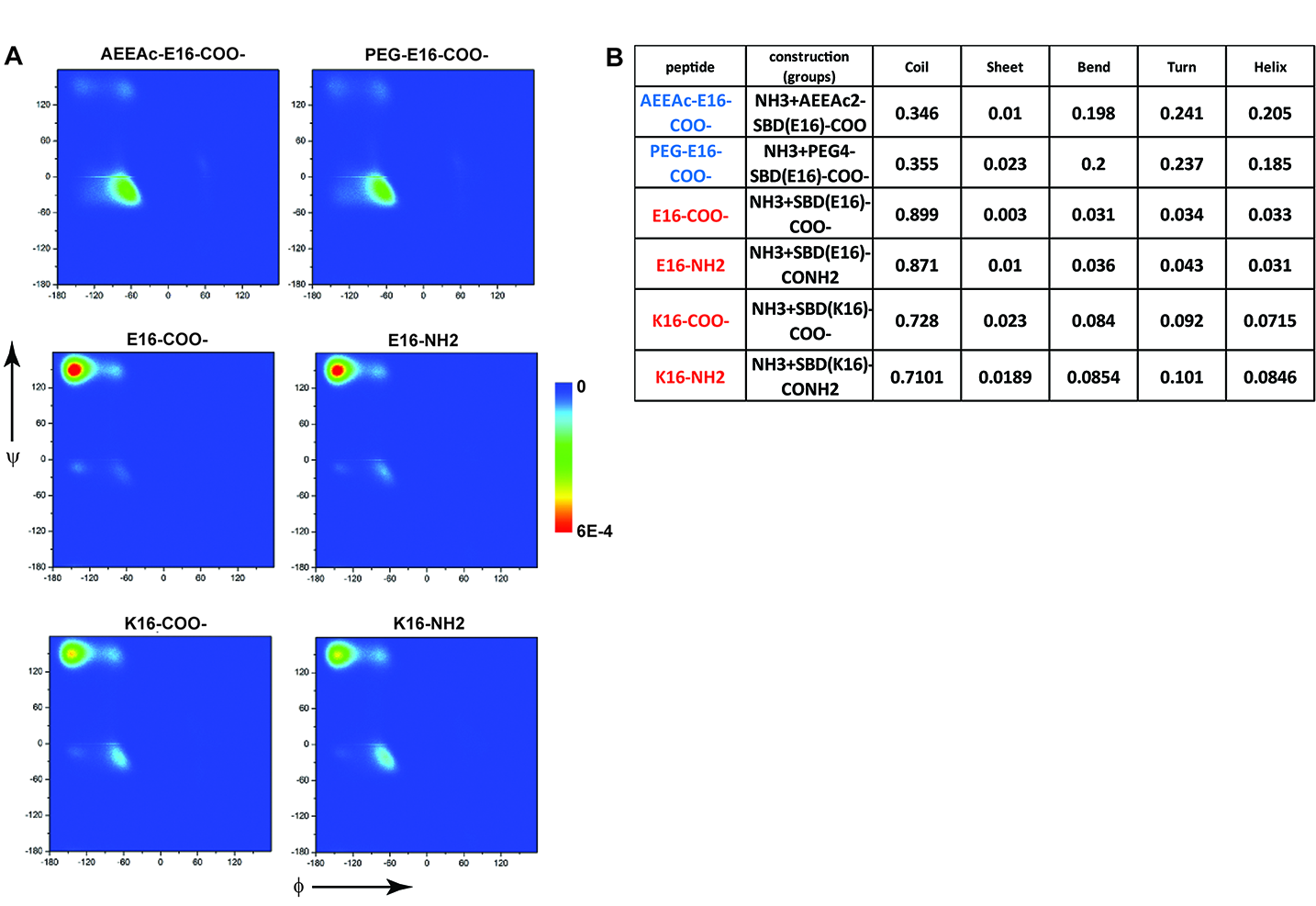

Supplement: Figure S2 — Ramachandran plots (A), with Phi (x-axis) and Psi (y-axis) showing the dihedral angles of the peptide backbone, which usually reflect the secondary structure; (B) secondary structure propensity (DSSP) analysis from 200 ns REMD simulations, with each number representing the fraction of the time that each structure occurs. The effect on SBD peptide structure was first compared between the AEEAc and the PEG linker. The distribution of structural features appeared to be very similar between the two. In the absence of linker, the distribution of structural features was compared between the four variants, either E16 or K16, and with carboxy (COO-) or amide (NH2) termini, in order to assess possible effects of these modifications on the structure in isolation. (TIF) [file pone.0051222.s002.tif]
